# IP_3_-mediated Ca^2+^ release regulates atrial Ca^2+^ transients and pacemaker function by stimulation of adenylyl cyclases

**DOI:** 10.1152/ajpheart.00380.2020

**Published:** 2020-10-16

**Authors:** Rebecca A. Capel, Samuel J. Bose, Thomas P. Collins, Skanda Rajasundaram, Thamali Ayagama, Manuela Zaccolo, Rebecca-Ann Beatrice Burton, Derek A. Terrar

**Affiliations:** ^1^Department of Pharmacology, British Heart Foundation Centre of Research Excellence, University of Oxford, Oxford, United Kingdom; ^2^Department of Physiology, Anatomy and Genetics, University of Oxford, Oxford, United Kingdom

**Keywords:** adenylyl cyclase, cardiac atria, Ca^2+^ signaling, cyclic AMP, inositol trisphosphate

## Abstract

Inositol trisphosphate (IP_3_) is a Ca^2+^-mobilizing second messenger shown to modulate atrial muscle contraction and is thought to contribute to atrial fibrillation. Cellular pathways underlying IP_3_ actions in cardiac tissue remain poorly understood, and the work presented here addresses the question whether IP_3_-mediated Ca^2+^ release from the sarcoplasmic reticulum is linked to adenylyl cyclase activity including Ca^2+^-stimulated adenylyl cyclases (AC1 and AC8) that are selectively expressed in atria and sinoatrial node (SAN). Immunocytochemistry in guinea pig atrial myocytes identified colocalization of type 2 IP_3_ receptors with AC8, while AC1 was located in close vicinity. Intracellular photorelease of IP_3_ by UV light significantly enhanced the amplitude of the Ca^2+^ transient (CaT) evoked by electrical stimulation of atrial myocytes (31 ± 6% increase 60 s after photorelease, *n* = 16). The increase in CaT amplitude was abolished by inhibitors of adenylyl cyclases (MDL-12,330) or protein kinase A (H89), showing that cAMP signaling is required for this effect of photoreleased IP_3_. In mouse, spontaneously beating right atrial preparations, phenylephrine, an α-adrenoceptor agonist with effects that depend on IP_3_-mediated Ca^2+^ release, increased the maximum beating rate by 14.7 ± 0.5%, *n* = 10. This effect was substantially reduced by 2.5 µmol/L 2-aminoethyl diphenylborinate and abolished by a low dose of MDL-12,330, observations which are again consistent with a functional interaction between IP_3_ and cAMP signaling involving Ca^2+^ stimulation of adenylyl cyclases in the SAN pacemaker. Understanding the interaction between IP_3_ receptor pathways and Ca^2+^-stimulated adenylyl cyclases provides important insights concerning acute mechanisms for initiation of atrial arrhythmias.

**NEW & NOTEWORTHY** This study provides evidence supporting the proposal that IP_3_ signaling in cardiac atria and sinoatrial node involves stimulation of Ca^2+^-activated adenylyl cyclases (AC1 and AC8) by IP_3_-evoked Ca^2+^ release from junctional sarcoplasmic reticulum. AC8 and IP_3_ receptors are shown to be located close together, while AC1 is nearby. Greater understanding of these novel aspects of the IP_3_ signal transduction mechanism is important for future study in atrial physiology and pathophysiology, particularly atrial fibrillation.

## INTRODUCTION

Ca^2+^ handling in the heart is vital to normal physiological function and regulation of excitation-contraction coupling (1, 2). Ca^2+^ signaling in cardiomyocytes is tightly regulated, for example, by protein kinase A (PKA) and Ca^2+^/calmodulin-dependent protein kinase II (CaMKII), or by Ca^2+^-mobilizing elements such as inositol trisphosphate (IP_3_), cADP-ribose (cADPR), and nicotinic acid adenine dinucleotide phosphate (NAADP), as well as by Ca^2+^ itself (1–3). The atrial and ventricular chambers of the heart have very different functions, and therefore it is not surprising that there are many differences between atrial and ventricular myocytes in excitation-contraction coupling and in the handling of Ca^2+^ ions by different intracellular compartments. One characteristic feature of atrial myocytes is the relative abundance of receptors for inositol trisphosphate (IP_3_) compared with ventricular myocytes (3). IP_3_ is a Ca^2+^-mobilizing second messenger (4) that acts to open IP_3_ receptors (IP_3_R), located on the sarcoplasmic reticulum (SR) of cardiomyocytes (3, 5). IP_3_ is positively inotropic in atrial (3) and ventricular (6) preparations and is positively chronotropic in the sinoatrial node (SAN) (7, 8). IP_3_ is synthesized upon stimulation of phospholipase C (PLC) commonly, but not exclusively, by G-protein coupled receptors associated with G_q_ (9). In cardiac myocytes, endothelin-1 (ET-1), angiotensin II (Ang-II), and phenylephrine (PE) all increase intracellular IP_3_ level (10) via their actions at the G_q_-coupled ET-A, Ang-II, and α-adrenergic receptors, respectively.

Early functional studies revealed a much greater effect of IP_3_-associated stimuli on the contractility of atrial preparations than upon their ventricular counterparts (11), and expression of IP_3_R type 2 (IP_3_R2) is now known to be at least six times greater in atrial myocytes (3). IP_3_R expression is significantly increased during atrial fibrillation in both human patients (12) and animal models (13). Inhibiting G_q_-coupled Ang-II receptors has been shown to prevent the early remodeling associated with rapid atrial pacing (14), whereas in healthy atrial myocytes, G_q_-associated signaling causes an IP_3_R-dependent increase in the Ca^2+^ spark rate of quiescent myocytes and amplitude of the stimulated Ca^2+^ transient (15), effects matched on direct application of IP_3_ [Ca^2+^ sparks (3, 16), Ca^2+^ transients (3)]. Interestingly, even in healthy cells, IP_3_-dependent stimulation can be associated with the generation of spontaneous diastolic Ca^2+^ events (3, 5).

Cardiac function is also controlled by pathways involving production of cAMP (1, 17). These pathways are commonly thought to be distinct and separate from the previously mentioned IP_3_-dependent mechanisms, although evidence supports a cAMP-mediated regulation of the sensitivity of IP_3_Rs to Ca^2+^ (18, 19). Here, we consider a possible novel link between cAMP-dependent mechanisms and IP_3_-mediated Ca^2+^ release from the SR, which arises from the existence and location of the Ca^2+^-stimulated isoforms of adenylyl cyclase, AC1 and AC8, which have been shown to be selectively expressed in SAN (20, 21) and atria (22), whereas in contrast, ventricular myocytes predominantly express AC5 and AC6 (23), which lack Ca^2+^ sensitivity. Interestingly, AC1 and AC8, like IP_3_R, have been shown to be expressed close to the surface of SA node and atrial myocytes, although the relative positions of these adenylyl cyclases and IP_3_Rs have not been previously explored (20).

Functionally, Ca^2+^-stimulated adenylyl cyclases have been shown to play a role in regulating pacemaker activity (8, 24), at least in part by controlling the synthesis of cAMP, which can directly regulate an important pacemaker current, I(f) (25), independently of basal PKA activity (26) and Ca^2+^/CAMKII-dependent phosphorylation (27). Expression of Ca^2+^-stimulated AC1 has also been shown to enhance beating rate in hyperpolarization-activated cyclic nucleotide-gated 2 (HCN2)-mediated “biological pacemakers” (28).

Previous experiments also provided evidence for a role for Ca^2+^-stimulated adenylyl cyclases in regulating Ca^2+^ transients and L-type Ca^2+^ currents in atrial myocytes (22). In particular, loading atrial myocytes with the Ca^2+^ chelator, BAPTA, was shown to cause a substantial reduction of the amplitude of L-type Ca^2+^ currents in atrial myocytes, but BAPTA was without effect when cytosolic cAMP levels were maintained at 200 μM by application from a patch pipette. These observations were interpreted as evidence for ongoing enhancement of L-type Ca^2+^ currents, which was dependent on basal activity of Ca^2+^-stimulated adenylyl cyclases in atrial myocytes. This interpretation was further supported by the observations that the amplitudes of L-type Ca^2+^ currents were also reduced when adenylyl cyclases were inhibited by MDL12-330A [cis-*N*-(2-phenylcyclopentyl)-azacyclotridec-1-en-2-amine hydrochloride] (22).

In view of the functional importance of Ca^2+^-stimulated adenylyl cyclases in regulating atrial and SAN function, and the likelihood that IP_3_Rs may be located close to AC1 and AC8, the hypothesis to be tested is that actions of IP_3_ in atria and SAN depend on IP_3_-mediated Ca^2+^ release from the SR leading to activation of the adenylyl cyclases, AC1 and AC8.

## METHODS

All animal experiments were performed in accordance with the United Kingdom Home Office Guide on the Operation of Animal (Scientific Procedures) Act of 1986. All experimental protocols (Schedule 1) were approved by the University of Oxford, Procedures Establishment License (PEL) Number XEC303F12.

### Atrial Myocyte Isolation

Male Dunkin Hartley guinea pigs (350–550 g, Envigo, UK) were housed and maintained in a 12-h light/dark cycle with ad libitum access to standard diet and sterilized water. Guinea pigs were culled by cervical dislocation in accordance with Home Office Guidance on the Animals (Scientific Procedures) Act (1986). Atrial myocytes were isolated following the method of Collins et al. (2011) (29) and stored at 4°C in a high potassium medium containing (in mmol/L): KCl 70, MgCl_2_ 5, K^+^ glutamine 5, taurine 20, EGTA 0.1, succinic acid 5, KH_2_PO_4_ 20, HEPES 5, glucose 10 at pH 7.2 with KOH. Healthy atrial myocytes were identified on the basis of morphology.

### Immunocytochemistry

Immunocytochemistry staining and analysis was carried out using the method of Collins and Terrar (2012) (22). AC1 (sc25743) and AC8 (sc32128) primary antibodies were purchased commercially (Santa Cruz Biotechnology, Santa Cruz, CA) and used at a dilution of 1:200. Specificity of sc25743 and sc32128 for AC1 and AC8 was confirmed by Western blot [methods and data previously published in Mattick et al. (20)]. IP_3_R monoclonal primary antibodies (IP_3_R1 KM1112, IP_3_R2 KM1083, IP_3_R3 KM1082) were a kind gift from Professor Katsuhiko Mikoshiba (30) and used at a dilution of 1:1,000. The specificity of antibodies KM1112, KM1083, and KM1082 has been previously verified using Western blot by Sugiyama et al. (31) as well as in previous publications (30). Use of these IP_3_R antibodies has been extensively covered in previous studies (32–35). All primary antibody staining was carried out overnight at 4°C. Secondary antibody labeling was carried out using either Alexa Fluor 488 or 555 conjugated secondary antibodies (Invitrogen, UK), raised against the appropriate species, for 60 min at room temperature at a dilution of 1:400. Observations were carried out using a Zeiss LSM 510 confocal microscope (×40 or ×63 oil objectives). For detection of Alexa Fluor 488, fluorescence excitation was at 488 nm with emission collected at 505–530 nm. An excitation filter of 543 nm and an emission filter at >560 nm were used to detect Alexa Fluor 555. The two channels were imaged sequentially. Control cells where the primary or secondary antibody was to be excluded were incubated with 5% donkey serum alone without addition of the relevant antibody. To quantify the relationship between the red and green signals that were imaged during double labeling experiments, we carried out a pixel-by-pixel colocalization analysis on whole cells in ImageJ [using the plugin “Just Another Co-localization Plugin” (36)]. The analysis assessed, pixel by pixel across the whole image, the correlation between intensity values of the two dyes viewed as grayscale images and used produced Pearson’s coefficient, which is between −1 (total exclusion of the signals) and +1 (complete colocalization of the signals).

### Ca^2+^ Transient Imaging and IP_3_ Photorelease

For whole cell fluorescence experiments, isolated atrial myocytes were incubated with Fluo-5F (3 µmol/L) for 10 min, then plated to a glass coverslip for imaging. Carbon fiber electrodes were used to field-stimulate Ca^2+^ transients at a rate of 1 Hz. All experiments were carried out at 35 ± 2°C (fluctuation within a single experiment was <0.5°C) under gravity-fed superfusion of physiological salt solution (PSS, in mmol/L): NaCl 125, NaHCO_3_ 25, KCl 5.4, NaH_2_PO_4_ 1.2, MgCl_2_ 1, glucose 5.5, CaCl_2_ 1.8, oxygenated with 95% O_2_-5% CO_2_ (solution pH 7.4 after oxygenation and heating). Solution flow rate was 3 mL/min.

For photorelease experiments, isolated atrial myocytes were incubated for 60 min at room temperature with 0.5 µmol/L membrane-permeant caged IP_3_ (caged-IP_3_/PM) and 0.025% pluronic F127 (Enzo Life Sciences, UK). Fluo-5F-AM (3 µmol/L) was added for the last 10 min of incubation. DMSO concentrations were 0.5% during IP3/PM loading and 0.75% during IP_3_/PM + Fluo-5F. Cells were visualized using a Zeiss Axiovert 200 with attached Nipkow spinning disk confocal unit (CSU-10, Yokogawa Electric Corporation, Japan). Excitation light, transmitted through the CSU-10, was provided by a 488-nm diode laser (Vortran Laser Technology Inc., Sacramento, CA). Emitted light was passed through the CSU-10 and collected by an iXON897 EM-CCD camera (Oxford Instruments, UK) at 60 frames per second with 2 × 2 binning (pixel size = 0.66667 µm^2^). UV uncaging was carried out using 3× rapid flashes of a Xenon arc lamp (Rapp Optoelectronics, Germany), delivered through the objective lens. To avoid dye bleaching, the cells were not continually exposed to 488 nm light. Instead, a video of 8–10 s of calcium transients was recorded at a number of timepoints immediately before photorelease (denoted 0 s) and at the indicated times after UV exposure. Calcium transients were measured using regions of interests (ROIs) in Andor iQ software (v 1.7) to record average whole cell fluorescence. For inhibitor work, each aliquot of IP_3_/PM (3–4 experiments) was first used for a control experiment and inhibitor data were excluded if control cells did not respond. Cells were also excluded if, upon analysis, control (pre-photorelease) data exhibited alternans, missed beats, or were otherwise unstable. Adenylyl cyclases were inhibited using the nonselective adenylyl cyclase inhibitor MDL 12–330 A (3 µmol/L) (37), whereas for inhibition of PKA, we used H89 (1 µmol/L). 1H-[1,2,4]Oxadiazolo[4,3-a]quinoxalin-1-one (ODQ; 10 µmol/L) and *N*^ω^-nitro-L-arginine methyl ester(L-NAME; 100 µmol/L) were used to inhibit soluble guanylyl cyclase and nitric oxide synthase, respectively. All inhibitors were sourced from Tocris Bioscience (UK), except L-NAME which was sourced from Sigma-Aldrich, and applied for at least 10 min, of which at least 5 min were stimulated at 1 Hz. Calcium transient time courses were analyzed in ClampFit (v 10.4) and rise and decay curve-fitting were carried out by fitting single exponentials using Prism (v 8).

### Murine Atrial Studies

Adult male CD1 mice (CD-1 IGS 30–35 g, Charles River, UK) were housed maintained in a 12-h light/dark cycle with ad libitum access to standard diet and sterilized water. Mice were culled by cervical dislocation in accordance with Home Office Guidance on the Animals (Scientific Procedures) Act (1986). The heart was rapidly excised and washed in heparin-containing PSS. The ventricles were dissected away under a microscope, and the area adjacent to the sinoatrial node was cleared of connective tissue. The spontaneously beating atrial preparation was mounted in a 37°C organ bath containing oxygenated PSS and connected to a force transducer (MLT0201 series, ADInstruments, UK) to visualize contractions. Resting tension was set between 0.2 and 0.3 g, the tension signal was low-pass filtered at 20 Hz, and beating rate was calculated from the time interval between contractions. After stabilization (variation in average rate of a 10-s sample of no more than 2 beats/min over a 10-min period), cumulative concentrations of PE were added to the bath (range 0.1–30 µmol/L) in the presence of metoprolol (1 µmol/L, applied 30 min before PE) to ensure specificity to α-adrenergic effects. Preparations were excluded if stabilized beating rate under control conditions (PSS only) was less than 300 beats/min or if preparations were not rhythmic. In addition to the inhibitors listed previously, IP_3_ receptors were inhibited using 2-aminoethyl diphenylborinate (2-APB) (2.5 µmol/L, Merck, UK) (37). AC1 was inhibited using the AC1 selective inhibitor ST034307 (1 µmol/L, Tocris, UK) (38), whereas U73122 (5 µmol/L, Tocris, UK) was used to inhibit IP_3_ production by PLC. Inhibitors were added after stabilization of the preparations and applied for either 30 min (2-APB, ODQ, L-NAME, U73122, and ST034307) or 60 min (MDL, H89) before PE additions. PE dose-response curves were started only after tissue had reached a stable response, where any occurred.

### Statistics

For all single-cell data, *t* tests or ANOVA were used as appropriate with Dunnett’s or Tukey’s post hoc test to compare groups to a single control or to all other groups, respectively, as required. Experimenters were not blinded to the conditions being analyzed. Log(concentration)-response curves, used to estimate EC_50_s and maximum responses, were calculated using Prism v8.4.0 software (GraphPad, CA), by fitting an agonist-response curve with a fixed slope to normalized response data. Normalized data were used to compare responses as it was expected some inhibitors used would significantly affect the control beating rate or Ca^2+^ transient amplitude. Fitted values were compared using ANOVA with Dunnett’s or Tukey’s post hoc test. For analysis of Ca^2+^ transient rise and decay times at 0 s and 120 s after IP_3_ photorelease, Ca^2+^ data were analyzed using pClamp v10 (Molecular Devices, CA) to generate times corresponding to 10%–90% and 10%–50% rise time and 90%-10%, 90%-75%, 90%-50%, and half-width decay time. Decay phases of transients were also fitted using one phase decay least squares regression (Prism v8.4.0). Data are presented as means ± SE of recorded values, other than dose-response curve maximum, which is given as means ± SE of best-fit value, and EC_50_, which is presented as best-fit value with 95% confidence interval.

## RESULTS

### Type 2 IP_3_ Receptors are Colocalized with AC8 in Cardiac Atrial Myocytes

**Figure 1.**
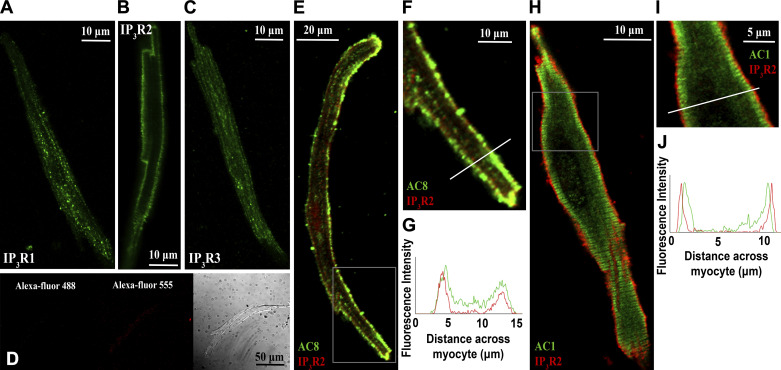
IP_3_R2 colocalizes with AC8 in guinea pig atrial myocytes. *A–C*: representative examples of fixed, isolated guinea pig atrial myocytes labeled for IP_3_R1 (*A*), IP_3_R2 (*B*), IP_3_R3 (*C*), and negative controls (*D*). *E*: representative example of a fixed, isolated guinea pig atrial myocyte co-immunolabeled for IP_3_R2 (red) and AC8 (green). *F*: digital zoom of the area indicated on image *D*. *G*: intensity plot to show staining intensity along the line shown in *E*. *H*: representative example of a fixed, isolated guinea pig atrial myocyte co-immunolabeled for IP_3_R2 (red) and AC1 (green). *I*: digital zoom of the area indicated on image *G*. *J*: intensity plot to show staining intensity along the line shown in *H*. IP_3_R1, inositol trisphosphate receptor type 1; IP_3_R2, inositol trisphosphate receptor type 2; IP_3_R3, inositol trisphosphate receptor type 3.

In agreement with published literature (3), type 2 IP_3_ receptors (IP_3_R2) were visualized in a punctate pattern at the cell periphery, consistent with a position on junctional SR (Fig. 1*B*). The vast majority of IP_3_R expression in cardiac atrial myocytes is thought to be IP_3_R2, with IP_3_R1 and IP_3_R3 as minimal components (3). Consistent with this notion, staining for IP_3_R1 (Fig. 1*A*) and IP_3_R3 (Fig. 1*C*) receptors did not demonstrate a distinct subcellular pattern and may represent at least some nonspecific labeling. Negative controls for immunocytochemistry (application of secondary antibody only) are shown in Fig. 1*D*.

Similar to previously published work in guinea pig SAN (20) and atrial myocytes (20, 22) and murine SAN (21), immunolocalization of AC8 indicated a band at or just beneath the sarcolemma. Pixel-by-pixel analysis revealed substantial colocalization between AC8 and IP_3_R2 in isolated guinea pig atrial myocytes, Pearson’s overlap coefficient *R* = 0.81 ± 0.02 (*n* = 14 cells), representative cell shown in Fig. 1, *E*–*G*.

AC1 staining was localized to a band which was consistently nearby but predominantly on the intracellular side of IP_3_R2 staining and signals were not substantially overlapping (*R* = 0.48 ± 0.05, *n* = 5, representative cell shown in Fig. 1, *H–J*). The pattern of AC1 and AC8 expression observed matched that for both AC1 and AC8 described previously in SAN cells (20).

### The Effect of IP_3_ on Cellular Ca^2+^ Transients Requires Functional Adenylyl Cyclases and PKA

**Figure 2.**
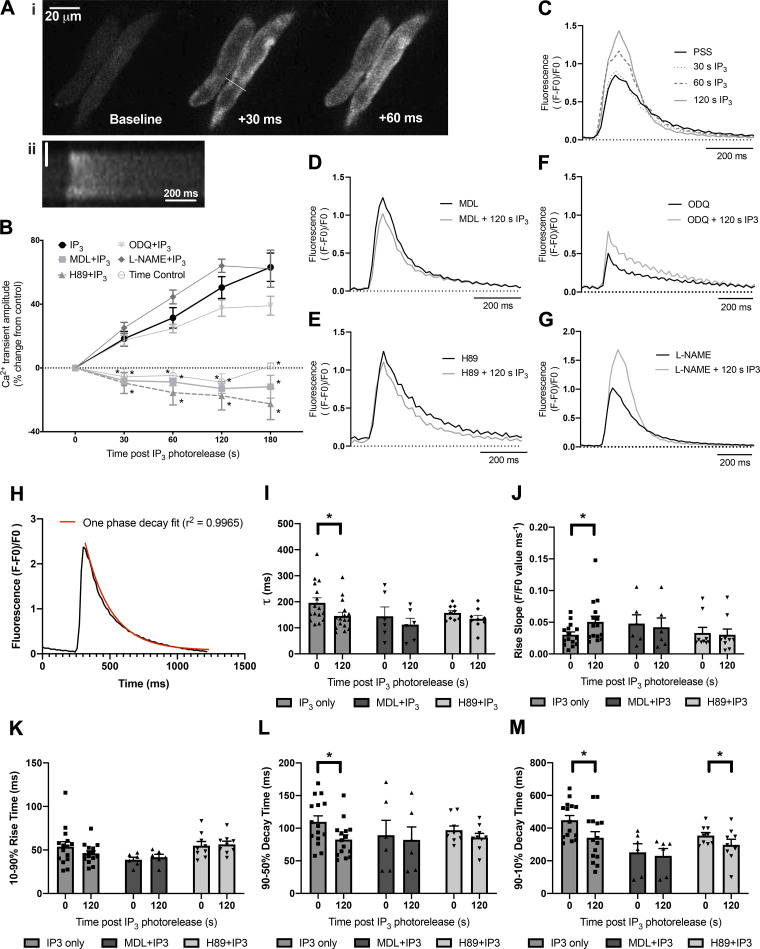
The direct actions of IP_3_ in guinea pig atrial myocytes require adenylyl cyclases and PKA. *Ai*: montage to show the upstroke of a control Ca^2+^ transient in PSS illustrating the classical U-shaped activation pattern. Numbers indicate time in ms from start of calcium transient upstroke. *Aii*: pseudolinescan image generated across the line drawn in *Ai* to show the U-shaped calcium transient seen in guinea pig atrial myocytes. Vertical scale bar indicates 5 µm. *B*: summary data to show cellular responses to IP_3_ photorelease (0.5 µmol/L caged-IP_3_/PM loaded for 1 h) in isolated guinea pig atrial myocytes under control conditions (*n* = 16) and in the presence of 1 µmol/L H89 (*n* = 9) to inhibit PKA, 3 µmol/L MDL (*n* = 6) to inhibit adenylyl cyclases, 10 µmol/L ODQ (*n* = 10) to inhibit soluble guanylyl cyclase, and 100 µmol/L L-NAME (*n* = 4) to inhibit nitric oxide synthase. *significant difference in comparison to IP_3_ photorelease alone (*P *<* *0.05, ANOVA with Dunnett’s post hoc test). *C*: representative Ca^2+^ transient response to photorelease of IP_3_ in PSS under control conditions and at indicated timepoints after photorelease. *D–G*: representative Ca^2+^ transients under control conditions and 120 s after photorelease of IP_3_ in MDL (*D*), H89 (*E*), ODQ (*F*), and L-NAME (*G*). *H*: representative Ca^2+^ trace showing transient decay period fitted using one phase decay equation. *I–M*: comparison of time constant of decay (τ) (*I*), maximum rise slope (*J*), 10%–90% rise time (*K*), 90-50% decay time (*L*), and 90-10% decay time (*M*) for all conditions. Data plotted as means ± SE, *P <* *0.05. IP_3_, inositol trisphosphate; IP_3_/PM, membrane-permeant caged IP3; PSS, physiological salt solution.

IP_3_ is not cell permeant and is broken down rapidly within cells. In addition, as activation of α-ARs (e.g., using PE) may result in signaling via alternative pathways including activation of PKC via diacylglycerol (DAG) (39), for our experiments, we used a cell-permeant, caged version of the compound (IP_3_/PM) to provide cell stimulation specifically via this second messenger from an exogenous source. This IP_3_ compound crosses the cell membrane, is de-esterified by constitutive esterase activity and trapped, and finally can be activated by “uncaging” through brief exposure to UV light. Exposure of cells to UV light alone under the conditions of this experiment did not affect calcium transient amplitude (Fig. 2*B*) or shape.

Guinea pig atrial myocytes exhibited the classical “U-shaped” activation pattern (40) of atrial myocyte Ca^2+^ transients (Fig. 2*A*, *i* and *ii*). Photorelease of IP_3_ in isolated cardiac atrial myocytes led to a gradual increase in stimulated Ca^2+^ transient amplitude (e.g., 31 ± 6% increase 60 s after photorelease, *n* = 16, Fig. 2, *B* and *C*. This response was completely abolished in the presence of either the adenylyl cyclase inhibitor MDL (3 µmol/L, *n* = 6, Fig. 2, *B* and *D*) or PKA inhibitor H89 (1 µmol/L, *n* = 9, Fig. 2, *B* and *E*), e.g., change in Ca^2+^ transient at 60 s after photorelease of −9 ± 2% in the presence of MDL and −16 ± 8% in the presence of H89. The control IP_3_ response was significantly greater than that of MDL or H89 at all measured timepoints after IP_3_ photorelease (*P* < 0.0002 for all comparisons except 30 s after photorelease in H89 where *P* = 0.0235, ANOVA with Tukey’s test for multiple comparisons), whereas the responses seen in the presence of MDL or H89 were not significantly different from one another, or time controls, throughout all timepoints (*P* > 0.85 for all comparisons).

As explained in the previous paragraph, our simple hypothesis is that the effects of photoreleased IP_3_ to increase CaT amplitude results from Ca^2+^ release from the SR which then activates Ca^2+^-stimulated adenylyl cyclases that are located nearby. However, a more complex hypothesis deserves consideration since PE responses in cat atrial myocytes have been reported to be dependent on nitric oxide-modulated soluble guanylyl cyclase activity by a mechanism that involves stimulation of NO synthase (eNOS) by IP_3_-mediated Ca^2+^ release from the SR (41). We therefore carried out IP_3_ photorelease in the presence of either 10 µmol/L ODQ to inhibit soluble guanylyl cyclase, or 100 µmol/L L-NAME to inhibit nitric oxide synthase (Fig. 2, *B*, *F,* and *G*). There was no change in the response to IP_3_ photorelease in the presence of ODQ (*P* > 0.56 for all timepoints, ANOVA, *n* = 10, Fig. 2, *B* and *F*) or L-NAME (*P* > 0.45 for all timepoints, ANOVA, *n* = 4, Fig. 2, *B* and *G*); under both conditions, Ca^2+^ transient amplitude increased significantly over time, beginning rapidly after photorelease of IP_3_ and was not significantly different to control at any timepoint (Fig. 2*B*). It therefore appears that under the conditions of our experiments, there is little or no contribution of the nitric oxide-stimulated guanylyl cyclase pathway to the effects of photoreleased IP_3_.

Further analysis of calcium transient characteristics at the 0 and 120 s timepoints (Fig. 2, *H–M* and Supplemental Fig. S1; all Supplemental material is available at https://doi.org/10.6084/m9.figshare.12333305) showed that photorelease of IP_3_ led to a significant increase in maximum upstroke velocity without affecting 10%–90% rise time (Fig. 2*J*) and a significant reduction in time constant of decay (Fig. 2*I*) as well as time to 90% and 50% recovery (Fig. 2, *L* and *M*). These changes were no longer seen in the presence of MDL. In the presence of H89, photorelease of IP_3_ had no significant effect on maximum upstroke velocity or time to 50% recovery, however time to 90% recovery was significantly reduced, though to a lesser extent than the effects seen after exposure to IP_3_ under control conditions (control; *P* < 0.0001; H89; *P* = 0.04).

Separate experiments were performed using external application of PE (10 µmol/L) to determine the effect of inhibition of adenylyl cyclases or PKA where alternative signaling pathways (e.g., PKC, DAG) may be involved (Supplemental Fig. S2). PE caused a 39 ± 10% increase in calcium transient amplitude (*n* = 6, *P* = 0.0006 PE condition vs. paired PSS control). As expected, PE no longer caused a significant increase in calcium transient amplitude when applied during inhibition of IP_3_Rs or α_1_-adrenergic receptors using 2-APB or prazosin, respectively (*P* = 0.961 for 2-APB and *P* = 0.998 prazosin condition vs. drug + PE condition, two-way ANOVA). In a separate set of experiments, PE caused a 35 ± 9% increase in peak-stimulated Ca^2+^ transient amplitude (*n* = 8). In the presence of adenylyl cyclase inhibitor MDL (10 µmol/L, *n* = 5) or PKA inhibitor H89 (1 µmol/L, *n* = 4), 10 µmol/L PE was no longer able to significantly enhance calcium transient amplitude (*P* = 0.689 for MDL and *P* = 0.137 for H89 condition vs. drug + PE condition, two-way ANOVA, Supplemental Fig. S2). This is consistent with the findings of the Blatter group working in cat atrial myocytes (41).

In assessing the importance of the above observations, it is necessary to take into account the influence of ongoing activity of adenylyl cyclases in the absence of stimulation by IP_3_-mediated Ca^2+^ release from the SR [described in Refs. (20) and (22)], as well as possible effects of cAMP on the IP_3_-evoked Ca^2+^ release (18, 42), and these points are considered in more detail in the discussion. Both MDL and H89 alone were previously shown (22) to reduce the amplitude and slow the time course of CaTs [MDL reduced CaT amplitude by 48 ± 8% (*P* < 0.001, *n* = 7), increased the time to peak by 45 ± 5% (*P* < 0.001, *n* = 7) and increased the time to 50% decay by 37 ± 13% (*P* < 0.05, *n* = 7), whereas H89 reduced CaT amplitude by 37 ± 5% (*P* < 0.01, *n* = 6), increased the time to peak by 19 ± 3% (*P* < 0.001, *n* = 6) and increased the time to 50% decay by 20 ± 6% (*P* < 0.05, *n* = 6)]. However, although CaT amplitude was reduced by both MDL and H89 because of ongoing adenylyl cyclase activity under resting conditions, there was still a substantial remaining CaT (greater than 50% peak amplitude), and if there were important effects of IP_3_-stimulated Ca^2+^ release from the SR that did not depend on adenylyl cyclases or downstream PKA effects then these would still be expected to operate and lead to observable increases in CaT amplitude under these conditions. The complete lack of any detectable increase in CaT amplitude following photorelease of IP_3_ in the presence of MDL or H89 therefore provides a clear indication for the requirement of adenylyl cyclase activity and downstream PKA to bring about the effects of IP_3_ in the absence of drugs. In a similar way, although some reduction of IP_3_-evoked Ca^2+^ release in the presence of MDL and PKA, as suggested from the work of Colin Taylor (18), cannot be excluded, the Ca^2+^ sensitivity of IP_3_Rs to IP_3_ would not be expected to be reduced to zero in the presence of MDL or H89, and therefore again the complete lack of detectable effects of photoreleased IP_3_ in the presence of MDL or H89 are attributable to the essential requirement of adenylyl cyclase activity and downstream PKA actions for the effects of IP_3_ in the absence of drugs.

### The Positive Chronotropic Effect of PE on the Sinoatrial Node Also Requires Functional Adenylyl Cyclases

**Figure 3.**
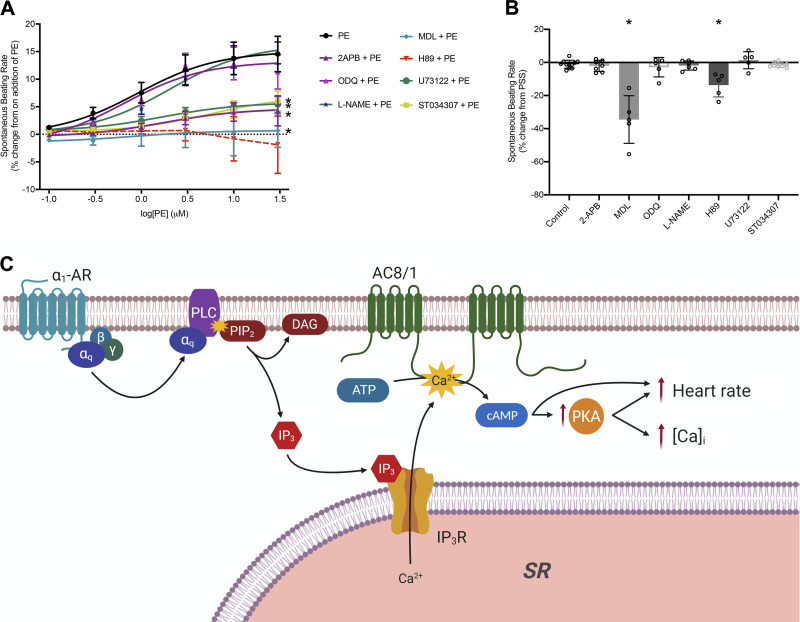
The positive chronotropic effect of PE requires function of adenylyl cyclases and a proposed scheme for regulation of intracellular calcium via IP_3_ signaling. *A*: dose-response curves to show the change in beating rate on cumulative addition of PE to spontaneously beating murine right atrial preparations under control conditions (*n* = 10) and in the presence of either 2-APB (IP_3_R inhibitor, 2.5 µmol/L, *n* = 7), MDL (AC inhibitor, 1 µmol/L, *n* = 5), ST034307 (AC1 inhibitor, 1 µmol/L, *n* = 8), H89 (PKA inhibitor, 1 µmol/L, *n* = 5), U73122 (PLC inhibitor, 5 µmol/L, *n* = 5), L-NAME (NOS inhibitor, 100 µmol/L, *n* = 6), or ODQ (sGC inhibitor, 30 µmol/L, *n* = 5). Dose-response curves (solid lines) were fit with log(agonist) versus response (three-parameter model) using GraphPad Prism 8. *significant reduction in maximum response of the fitted curve by ANOVA in comparison to PE. *B*: comparison of beating rate change in spontaneously beating murine atrial preparations from stable beating in PSS on addition of the inhibitors used in *A*, prior to stimulation by PE. Control indicates addition of β-blockade only as a time control (1 µmol/L metoprolol, *n* = 10). Other conditions are β-blockade plus indicated inhibitor. *P <* *0.001 compared to control (one-way ANOVA with Dunnett’s post hoc correction). Data plotted as means ± SE. *C*: scheme indicates potential mechanisms by which activation of α1 adrenergic receptors (α1-AR) by PE may lead to increased atrial cytoplasmic calcium transients (indicated by [Ca^2+^]_i_) and sinoatrial node beating rate based on published observations in addition to our present results. Activation of α1-AR leads to elevated IP_3_ resulting from cleavage of PIP_2_ to DAG and IP_3_ by PLC. IP_3_ activation of IP_3_R2 results in release of Ca^2+^ from the SR, which subsequently leads to activation of Ca^2+^-sensitive adenylyl cyclases (AC8 or AC1) and activation of PKA by cAMP, or direct effects of cAMP on the funny current I(f). In the proposed scheme, AC8 is placed in the sarcolemma, but it remains to be established whether there is an additional location in the junctional SR and whether nearby AC1 may also be activated by IP_3_-mediated Ca^2+^ release. Image created with BioRender. DAG, diacylglycerol; IP_3_, inositol trisphosphate; IP_3_R2, inositol trisphosphate receptor type 2; PE, phenylephrine; PSS, physiological salt solution; SR, sarcoplasmic reticulum; 2-APB, 2-aminoethoxydiphenyl borate.

It has been established that endogenous generation, or exogenous administration, of IP_3_ in the SAN leads to an increase in spontaneous beating rate, accompanied by an increase in Ca^2+^ transient amplitude (7), whereas cAMP from Ca^2+^-stimulated adenylyl cyclases has been shown to modulate murine heart rate (21) and, specifically, I(f) in these cells (20). Spontaneously beating atrial tissue preparations can also provide a measure of sinoatrial node activity through measurement of beating rate. IP_3_R2 (7) and AC8 (21) expression have previously been demonstrated in murine sinoatrial node, with that of AC8 highly similar to data presented in Fig. 1 and that of IP_3_R2 including both peripheral staining as seen in atrial myocytes (e.g., Fig. 1) and separate bands on non-junctional SR. Our own observations (20) demonstrate that AC1 and AC8 appear to show similar distribution within SAN cells as atrial cells. Dose-response curves to PE in the concentration range 0.1–30 µmol/L were carried out on spontaneously beating isolated murine right atria in the presence of 1 µmol/L metoprolol to ensure no confounding action of β-adrenergic receptors and fit with Log(agonist) versus response curves (three-parameter model) by nonlinear regression using a least squares method (Prism 8). Preparations were allowed to reach a stable beating rate in PSS and cumulative addition of PE then took place after either 30 min of metoprolol exposure (used as a time-control for the effect of other inhibitors) or exposure to metoprolol plus named inhibitor. Under these conditions, the positive chronotropic response to PE fits a standard agonist dose-response curve with an EC_50_ of 0.91 µmol/L [95% confidence interval (CI) 0.68–1.21] and a maximum rate increase of 15.1 ± 0.2% (*n* = 10, Fig. 3*A*).

Addition of a low concentration of 2-aminoethyl diphenylborinate (2-APB, 2.5 µmol/L), which is low enough to inhibit IP_3_-dependent effects in cardiomyocytes without altering cellular Ca^2+^ transient amplitude or SERCA function (7, 37, 43, 44), had no significant effect on right atrial beating rate over the course of at least 30 min (*P* = 0.9995, one-way ANOVA with Dunnett’s correction vs. time-control, *n* = 7, Fig. 3*B*). In the presence of 2-APB, the maximum rate increase on addition of PE was significantly reduced to 4.7 ± 0.2% (*P* < 0.0001 vs. PE, ANOVA with Dunnett’s correction, *n* = 7, Fig. 3*A*, EC_50_ 1.69 µmol/L, 95% CI 0.99–2.89). In agreement with this, 5 µmol/L U73122 to inhibit IP_3_ production by inhibition of phospholipase C (PLC) caused no significant change in beating rate (1.3 ± 2.3%, *P* = 0.9687 vs. time-control, *n* = 5, Fig. 3*B*) and significantly reduced the maximum response to PE (to 5.8 ± 0.1%, *n* = 5, *P* < 0.0001 vs. PE, ANOVA, Fig. 3*A*, EC_50_ 1.67 µmol/L, 95% CI 1.19–2.35).

In the presence of 1 µmol/L MDL to inhibit adenylyl cyclase activity, we observed a 34.5 ± 6.4% reduction in beating rate in the absence of further intervention (Fig. 3*B*, *P* < 0.0001, one-way ANOVA with Dunnett’s correction vs. time-control, *n* = 5). Under these conditions, bath application of cumulative doses of PE no longer led to an increase in beating rate (maximum rate change 0.7 ± 0.2%, *n* = 5, Fig. 3*A*) without significant effect on EC_50_ (0.85 µmol/L, 95% CI 0.08–7.63). Application of 1 µmol/L ST034307, a selective inhibitor of AC1 (8), led to a significant reduction in the effect of PE (*P* < 0.0001 vs. PE, maximum response from dose-response curve fit 7.0 ± 0.3%, *n* = 8, Fig. 3*A*) and a significant shift in EC_50_ (to 5.60 µmol/L, 95% CI 3.73–9.64, *P* = 0.0163 vs. response to PE, ANOVA with Dunnett’s correction) without a significant change in initial beating rate (Fig. 3*B*, *P* = 0.9998). Application of 1 µmol/L H89 to inhibit PKA also led to a significant reduction in beating rate (by 13.7 ± 3.2%, *P* = 0.0014 vs. time-control, *n* = 5, Fig. 3*B*) and completely abolished the effect of PE (largest measured rate change, at 30 µM PE, −1.9 ± 5.2%, *n* = 5, Fig. 3*A*). In the presence of H89, it was no longer possible to accurately fit a dose-response curve using the same model, it is therefore excluded from the statistical analyses based on curve fitting.

In agreement with the IP_3_ photorelease data, neither L-NAME (100 µmol/L, *n* = 6), nor ODQ (30 µmol/L, *n* = 5) had a significant effect upon spontaneous beating rate under control conditions, the maximum response to PE or EC_50_ (Fig. 3, *A* and *B*).

## DISCUSSION

The present observations indicate the need for an important extension to the proposed signaling pathways underlying the well-recognized actions of IP_3_ in atria and SAN and provide evidence for an alternative to the previous hypotheses that Ca^2+^ released from the SR via IP_3_ receptors may increase the amplitude of Ca^2+^ transients as a direct result of priming nearby ryanodine receptors (RyRs) (3) or by activating of eNOS located in caveolae in the surface membrane (41). This study represents the first measurements that link direct cellular stimulation with IP_3_ in atrial myocytes to downstream actions via the generation of cAMP and activation of PKA. Our work is consistent with the hypothesis that interaction of IP_3_-mediated Ca^2+^ release with the cAMP system is essential for the positive inotropic and chronotropic effects of this compound in the cardiac atria and sinoatrial node, and that this is physiologically important in the response of these tissues to α-adrenoceptor stimulation. The generation of cAMP by adenylyl cyclases in this newly proposed IP_3_-dependent pathway will itself have functional effects in SAN by increasing the activation of I(f) (20), and subsequent stimulation of PKA will have well-established downstream effects on phospholamban/SERCA (45), L-type Ca^2+^ channels (22), and perhaps RyR (46) in both SAN and atria. Structural studies using immunostaining methods (Fig. 1), which initially led us to investigate this intriguing possibility within our preparations, highlight the Ca^2+^-stimulated isoforms AC8 and AC1 as probable candidates for this interaction.

Before considering subtypes of adenylyl cyclase in more detail, our proposal of a direct involvement of Ca^2+^-stimulated adenylyl cyclases in the actions of IP_3_ in atria and SA node should be set in the broader context of interactions between IP_3_ and cAMP pathways. A particularly interesting possibility is that cAMP influences IP_3_-evoked Ca^2+^ release sensitivity to Ca^2+^ (42). It has been shown that IP_3_Rs, including IP_3_R2, can be phosphorylated by PKA enhancing IP_3_-evoked Ca^2+^ release (18, 42). In addition, high concentrations of cAMP can cause PKA-independent modulation of IP_3_Rs (18, 42). We cannot exclude the possibility that such mechanisms also operate in atria and SA node under the conditions of our experiments, but as set out in the results, we do not believe that such a possibility invalidates our use of MDL and H89 to investigate the requirement of adenylyl cyclases for the actions of IP_3_ in these tissues. We argue that substantial (greater than 50% peak amplitude) CaTs remain in the presence of MDL and H89, and even if the IP_3_-evoked Ca^2+^ release were to be reduced in the presence of MDL or H89, this IP_3_-evoked Ca^2+^ release would not be expected to be reduced to zero. The suggestion that IP_3_ receptors remain functional in the absence of cAMP or phosphorylation is based on extensive published work on regulation of IP_3_R2 receptors, and in all cases, it has been observed that these receptors continue to be activated by IP_3_ in the absence of cAMP or phosphorylation by PKA (18, 42, 47, 48). In the case of phosphorylation of IP_3_R2, the extent of enhancement of Ca^2+^ release has been observed to vary with IP_3_ concentration, and at 1 µM [see Fig. 2*C* in (47)], there was no detectable change in the sensitivity of IP_3_R2 to IP_3_ following PKA effects. Since no previous work is consistent with the proposal that IP_3_ receptors are unresponsive to IP_3_ in the absence of cAMP or without phosphorylation, we argue that our observation of a complete suppression of the effects of IP_3_ photorelease on CaTs in the presence of MDL and H89 provides convincing evidence supporting the proposal that, at least in atria, IP_3_-evoked Ca^2+^ release from the SR activates Ca^2+^-sensitive adenylyl cyclases and downstream PKA to bring about an enhancement of CaT amplitude. Similar arguments can be advanced for suppression of PE effects on beating rate in SA node, but in this case, additional complications that need to be taken into account are direct effects of cAMP (inhibited by MDL but not H89) and possible roles of DAG that are activated via α receptors in addition to IP_3_ release, and future experiments will be necessary to further test these possibilities.

Ten mammalian adenylyl cyclase isoforms have been discovered, nine membrane bound and one soluble form. Of these, three are Ca^2+^ sensitive; AC1 is CaM dependently Ca^2+^ stimulated (49) with an EC_50_ for Ca^2+^ of 75 nmol/L (50), AC8 is CaM dependently stimulated (51, 52) with a Ka for Ca^2+^ activation of ∼0.5 µmol/L (53) and AC5 is CaM-independently inhibited (54, 55). The majority of previous studies on AC1 and AC8 pertain to roles in the brain, where these enzymes have been implicated in a range of processes including spatial memory formation (56–58), neurodevelopment (59), responses to inflammatory pain (60), and opioid dependence (61). AC1 may also have a role in podocytes of the glomerulus of the kidney (62). Our immunocytochemistry data demonstrate that AC8 is found in close proximity to IP_3_Rs in cardiac atrial myocytes, whereas AC1 is found in a band just intracellular to these receptors. AC8, therefore, is ideally positioned to transduce local changes in Ca^2+^ into the cAMP-dependent and PKA-dependent effects detailed in this paper, namely, the modulation of cellular Ca^2+^ transients in response to IP_3_. Given the known position of IP_3_Rs on the junctional SR (3, 5), it is not possible for our staining to distinguish whether AC8 is located on the SR itself or on the surface membrane, situated less than 20 nm away (63, 64). Sucrose-based fractionation of isolated SAN myocytes has indicated that AC1 and AC8 activity is most associated with fractions also containing caveolin-3 (65). In other cell types, AC8 has been localized to caveolae (66), and disruption of lipid rafts has been seen to abolish the stimulation of this cyclase by Ca^2+^ (53). Taken together, this evidence is consistent with a surface membrane distribution of this enzyme. Although it seems most likely that Ca^2+^ released via IP_3_Rs activates colocalized AC8, the selective AC1 inhibitor ST034307 (38) significantly attenuated the response of whole atria beating rate to PE (Fig. 3*A*), raising the possibility that this Ca^2+^ could also activate nearby AC1.

The schematic shown in Fig. 3*C* provides an illustration of the cellular pathway, supported by the data presented in this paper, which may result following activation of the IP_3_ signaling cascade. Increased generation of cAMP via Ca^2+^ activation of AC8 or AC1 may lead to activation of PKA and multiple downstream actions that result in increases in cellular Ca^2+^ transient amplitude or beating rate at the sinoatrial node. AC1 and AC8 expression has previously been demonstrated in the SAN and these AC isoforms have been implicated in the regulation of SAN pacemaker activity (20, 21). Moen et al. (2019) (21) recently demonstrated that overexpression of AC8 in SAN cells results in an increase in heart rate and decrease in heart rate variability. Furthermore, work from the Terrar group has previously demonstrated the involvement of Ca^2+^-sensitive ACs in the regulation of I(f) in SAN myocytes (20) as well as L-type Ca^2+^ current in atrial myocytes (22). Taken together, these previous studies implicate both LTCC and HCN4 as potential targets for regulation via IP_3_ signaling in the context of the data presented in Figs. 2 and 3. The involvement of other targets for PKA phosphorylation however, including ryanodine receptors (RyRs) (46), phospholamban (PLB) (45), and the Na^+^/Ca^+^ exchanger (NCX) (67, 68) cannot yet be discounted. It is possible that the activity of PKA augments regulation by PKC, which has also been well documented at these same target sites and is similarly activated via stimulation of G_q_-coupled receptors (24, 69–72). The use of caged-IP_3_ in this study however rather than stimulation of G_q_-coupled receptors (Fig. 2) demonstrates that the effects on cellular Ca^2+^ observed in the present study can occur via the effects of IP_3_ signaling specifically and are independent of activation of DAG.

Under the conditions of our study, inhibition of ACs or PKA significantly reduced baseline beating rate in right atrial preparations (Fig. 3*B*). This is consistent with published data from our group (20, 22) and others (73, 74). Indeed, it has previously been shown that heart rate in AC8 overexpressing mice is significantly higher than in their wild-type counterparts (75). The sinoatrial node has a constitutive level of cAMP, which is significantly greater than that of the ventricle in the absence of adrenergic stimulation (26). How much of this activity is attributable specifically to Ca^2+^-stimulated ACs is not discernable from our experiments, as selective inhibitors are not currently available for all ACs, although 1 μmol/L ST034307 did not affect baseline rate (Fig. 3). The diastolic cell Ca^2+^ concentration in SAN myocytes, ∼225 nmol/L (76), is considerably higher than that in ventricular cells. Given that AC1 and AC8 proteins have not been shown to be expressed in ventricular tissue (20), it seems possible that differences in the expression of Ca^2+^-stimulated AC isoforms could contribute to the differences between resting SA nodal and ventricular cAMP concentration as previously reported (26). Indeed, cAMP synthesis activity is high in SAN myocyte lysates in 1 µmol/L Ca^2+^ but almost abolished in Ca^2+^-free solution (65), suggesting Ca^2+^-stimulated cAMP production may be the dominant mechanism in these cells at rest.

Our data indicate that high constitutive cAMP production in the sinoatrial node cannot be attributed to background IP_3_R activity. Background activity under the conditions of our experiments appears to be negligible as neither 2-APB, nor U73122 had a significant effect on the spontaneous beating rate of intact right atrial preparations when applied in our organ bath setup. This is in contrast to published work from Ju et al. (2011) (7) and Kapoor et al. (2015) (8), who both report significant reductions in spontaneous Ca^2+^ transient firing rate in response to 2-APB measured using Ca^2+^ fluorophores in dissected nodal tissue and isolated sinoatrial node myocytes, respectively.

In atrial myocytes isolated from cat, the effects of PE to enhance I_CaL_ have been reported to occur through inhibition of phosphodiesterase downstream of PI-3K-mediated eNOS activation (41). Although we agree that cAMP and PKA are central to the response of atrial myocytes and the sinoatrial node to PE, and IP_3_, we did not find evidence that nitric oxide or soluble guanylyl cyclase activity were required under the conditions of our experiments.

It has previously been hypothesized that Ca^2+^ release through IP_3_Rs acts to enhance atrial myocyte Ca^2+^ transients by increasing the local Ca^2+^ concentration around RyRs and thereby enhancing RyR response to the opening of LTCC (3, 16). Although this has been directly observed in an IP_3_R overexpression model (77), our data investigating the effect of IP_3_ photorelease in primary isolated myocytes is consistent with the notion that the functional effects of IP_3_R-mediated Ca^2+^ release on RyRs occur downstream of intermediary signals. In particular, it would be expected that recruitment of local RyRs in response to photoreleased IP_3_ would remain after inhibition of adenylyl cyclases or PKA. We observed no change in Ca^2+^ transient amplitude over time in response to direct application of IP_3_ in the presence of MDL or H89. It remains possible that RyR sensitization resulting from localized Ca^2+^ release via IP_3_Rs is essential in the period before our measurements take place, or that augmented RyR release could itself contribute to recruitment of adenylyl cyclase and PKA signaling.

The results of this study provide a novel mechanism by which a ubiquitous second messenger pathway contributes to physiological signaling in the heart. The presented hypothesis, however, may also provide interest in the context of cardiac pathology. For instance, Mougenot et al. (2019) (78) have recently demonstrated that overexpression of AC8 accelerates age-related cardiac dysfunction through increased hypertrophy and interstitial fibrosis in transgenic mice. Even in healthy atrial myocytes, IP_3_R signaling can generate arrhythmogenic Ca^2+^ waves (3, 5). The IP_3_ pathway may contribute to spontaneous generation of action potentials in pulmonary vein sleeve cells (79, 80), one of the main sites for AF initiation (81), whereas IP_3_R is known to be upregulated in atrial myocytes from patients with chronic AF (12) and from animal models of atrial fibrillation (AF) (13). In fact, atrial IP_3_R expression appears to correlate with markers of atrial dysfunction regardless of diagnosed disease state (12). In contrast, RyR expression becomes downregulated in chronic AF (82) and the organization of RyR clusters is disrupted (83). The relative time course of these expression changes during disease development is unknown. The position and character of the signaling domains presented in this paper remain to be determined in the context of the remodeling associated with AF, though the evidence presented in this paragraph highlights the importance of understanding the cellular mechanisms of the IP_3_ pathway and role of Ca^2+^-sensitive ACs in both healthy and diseased cardiomyocytes.

The present paper provides novel information on the signaling pathways responsible for physiological responses to IP_3_, demonstrating a crucial requirement for cAMP and PKA. We have focused on Ca^2+^-stimulated ACs as an effector of this interaction. Our new observations provide an added level of complexity to Ca^2+^ modulation in the atria and sinoatrial node and raise questions concerning the importance of these interacting signaling pathways in atrial fibrillation and related pathology.

## GRANTS

This work is supported by Sir Henry Dale Wellcome Trust and Royal Society Fellowship (109371/Z/15/Z; to R.A.B.B.). This project was supported by a British Heart Foundation Project Grant (PG/18/4/33521). R.A.C. is a Post-doctoral Scientist funded by the Wellcome Trust and Royal Society (109371/Z/15/Z). T.P.C. was funded through a British Heart Foundation DPhil studentship (FS/05/121) in the D.A.T. lab. S.J.B. is a Post-doctoral Scientist funded by the British Heart Foundation (PG/18/4/33521). T.A. received funding from the Returners Carers Fund (PI R.A.B.B.), Medical Science Division, University of Oxford, the Nuffield Benefaction for Medicine and the Wellcome Institutional Strategic Support Fund, University of Oxford.

## DISCLOSURE

No conflicts of interest, financial or otherwise, are declared by the authors.

## AUTHOR CONTRIBUTIONS

R.A.B.B. and D.A.T. conceived and designed research; R.A.C., S.J.B., T.P.C., S.R., and T.A. performed experiments; R.A.C., S.J.B., and T.P.C. analyzed data; R.A.C., S.J.B., M.Z., R.A.B.B., and D.A.T. interpreted results of experiments; R.A.C., S.J.B., and T.P.C. prepared figures; R.A.C., R.A.B.B., and D.A.T. drafted manuscript; R.A.C., S.J.B., T.P.C., S.R., M.Z., R.A.B.B., and D.A.T. edited and revised manuscript; R.A.C., S.J.B., M.Z., R.A.B.B., and D.A.T. approved final version of manuscript.
